# Impaired parasympathetic function in long-COVID postural orthostatic tachycardia syndrome – a case-control study

**DOI:** 10.1186/s42234-023-00121-6

**Published:** 2023-09-06

**Authors:** Stefano Rigo, Vasile Urechie, Andrè Diedrich, Luis E. Okamoto, Italo Biaggioni, Cyndya A. Shibao

**Affiliations:** 1 Department of Clinical Pharmacology, Vanderbilt University Medical Center, Nashville, TN USA; 2 Department of Biomedical Sciences, Humanitas University, Via Rita Levi Montalcini 4, Pieve Emanuele, Milan, 20090 Italy; 3 Department of Biomedical Engineering, Vanderbilt University, Nashville, TN USA

**Keywords:** Long-COVID, POTS, ANS, Parasympathetic, HRV, Spectra

## Abstract

**Purpose:**

Eighty percent of patients infected by SARS-CoV-2 report persistence of one symptom beyond the 4-week convalescent period. Those with orthostatic tachycardia and orthostatic symptoms mimicking postural tachycardia syndrome, they are defined as Long-COVID POTS [LCP]. This case-control study investigated potential differences in autonomic cardiovascular regulation between LCP patients and healthy controls.

**Methods:**

Thirteen LCP and 16 healthy controls, all female subjects, were studied without medications. Continuous blood pressure and ECG were recorded during orthostatic stress test, respiratory sinus arrhythmia, and Valsalva maneuver. Time domain and power spectral analysis of heart rate [HR] and systolic blood pressure [SBP] variability were computed characterizing cardiac autonomic control and sympathetic peripheral vasoconstriction.

**Results:**

LCP had higher deltaHR (+ 40 ± 6 vs. + 21 ± 3 bpm, *p* = 0.004) and deltaSBP (+ 8 ± 4 vs. -1 ± 2 mmHg, *p* = 0.04) upon standing; 47% had impaired Valsalva maneuver ratio compared with 6.2% in controls (*p* = 0.01). Spectral analysis revealed that LCP had lower RMSSD (32.1 ± 4.6 vs. 48.9 ± 6.8 ms, *p* = 0.04) and HF_RRI_, both in absolute (349 ± 105 vs. 851 ± 253ms^2^, *p* = 0.03) and normalized units (32 ± 4 vs. 46 ± 4 n.u., *p* = 0.02). LF_SBP_ was similar between groups.

**Conclusions:**

LCP have reduced cardiovagal modulation, but normal sympathetic cardiac and vasoconstrictive functions. Impaired parasympathetic function may contribute to the pathogenesis of Long-COVID POTS syndrome.

## Introduction

The COVID-19 pandemic has caused unprecedented morbidity and mortality worldwide [1, 2]. According to CDC, over 146 million people have been infected and about 1 million died so far in the US alone. The causative agent is SARS-CoV-2, an enveloped, positive-sense RNA virus able to infect the oropharyngeal mucosa, the lower respiratory tract epithelium, the myocardium [3], the endothelium [4], the immune system [5] and the nervous system [6, 7] inducing damages in multiple organ systems [8, 9].

Up to 80% of patients infected by SARS-CoV-2 develop chronic, disabling symptoms [10], including fatigue, chest pain, reduced exercise tolerance, tachycardia and cognitive impairment, even after mild or asymptomatic COVID-19 [11]. This syndrome, which persists after resolution of the acute infection, was named “Long-COVID”, “Post-Acute COVID-19 Sequelae” or “Post-Acute COVID19 Syndrome” [PACS] [12–15]. Given that over 140 million people in the US had COVID-19 infection, there is a growing healthcare concern [16, 17].

Persistent tachycardia remains one of the most common and disabling symptoms, reported from 9% up to 62% of COVID-19 survivors [18–21]. Positional tachycardia and chronic orthostatic intolerance symptoms in PACS mimic Postural Orthostatic Tachycardia Syndrome [POTS] [22, 23], a condition that is associated with autonomic nervous system dysfunction and that can be triggered after an acute viral illness [24–27]. Because of these similarities and because of the growing number of case reports [16, 28–30] the term Long-COVID POTS [LCP] was coined for PACS patients who met the diagnostic criteria for POTS [21, 31, 32].

Autonomic changes after SARS-CoV-2 infection are under investigation. Alterations in sympathovagal balance including increased cardiac sympathetic modulation have been described in PACS patients using Power Spectral Analysis techniques [33]. Elevated resting muscle sympathetic nerve activity was reported in COVID-19 infected subjects without residual symptoms [34], and around 15% of patients who recovered from mild to moderate SARS-CoV-2 pneumonia were found to have decreased parasympathetic cardiac control [35]. However, Heart-Rate Variability [HRV] was reported both to be increased [34, 36] and reduced [28, 33] in PACS patients. This likely reflect the heterogeneity of PACS and the different pathophysiological mechanisms in place, resulting in different autonomic profiles. Severity of disease, hospital stay, comorbidities and concomitant deconditioning from prolonged bedrest may all play a role and act as confounders for autonomic profile determination. On the other hand, patient with a specific manifestation of PACS, the excessive orthostatic tachycardia and/or orthostatic intolerance, likely share common etiology.

Of note, a detailed characterization of autonomic functions of LCP is not yet available. The specific mechanisms underlying LCP remain unknown, and it is unclear whether the autonomic nervous system is compromised in these patients.

## Methods

The aim of the case-control study here presented was to determine differences in cardiac sympathovagal modulation and sympathetic peripheral vascular control between LCP patients and controls.

### Study populations

LCP patients were identified from referrals to the Vanderbilt Autonomic Dysfunction Center from March to December 2021. Diagnosis was established after confirmation of all the following inclusion criteria: (1) previous real-time-PCR diagnosis of SARS-CoV-2 infection; (2) new onset of orthostatic intolerance after the infection and persistent symptoms for more than three months; and (3) orthostatic tachycardia > 30 beats-per-minute within 10 min of standing without concomitant orthostatic hypotension [32, 37]. Subjects were excluded if they were pregnant or breastfeeding; if they were obese (BMI > 30); if they had a history of diabetes mellitus, chronic kidney disease, liver disease or a previous diagnosis of autonomic dysfunction, neurodegenerative or cardiovascular disease; if they had previous history of POTS or orthostatic intolerance; if they had severe COVID-19 disease at presentation, defined as presentation respiratory rate > 30 breaths/min, SpO2 < 94% on room air at sea level, PaO2/FiO2 < 300mmHg or lung infiltrates > 50% [38] or prolonged hospital stay (> 30 days of hospitalization) due to COVID-19.

A population of healthy controls was enrolled for comparison. These subjects were recruited in pre-pandemic period, from August 2019 to March 2020, using flyers or massive email advertisements. They were young, non-obese female not affected by diabetes mellitus, chronic kidney disease, liver disease, neurodegenerative or cardiovascular disease and were not actively looking for a consult at the Autonomic Dysfunction Center clinic. Further, being studied in pre-pandemic period, they were not exposed to SARS-CoV-2.

Subjects were asked to fast and abstain from exercise at least 12 hours before studies, and all pharmacological agents with antiarrhythmic and autonomic effects were withheld for at least 72 hours before the study session. All subjects had a medical history and physical exam performed by an autonomic specialist. This study was approved by an institutional review board (IRB#220,550, PI C. A. Shibao & IRB#190,703, PI A. Diedrich) and all participants gave written informed consent. This study was conducted under VUMC institutional guidelines and adhered to the principles of the Declaration of Helsinki and Title 45 of the US Code of Federal Regulations (Part 46, Protection of Human Subjects).

### Study protocol

All subjects underwent an initial orthostatic vital sign assessment, during which blood pressure and heart rate were obtained in different positions; once after lying supine for at least 5 min, once after 5 min of sitting with their feet on the floor and once after standing for 10 min, using an automated arm-cuff blood pressure device (Vital-Guard 450 C, Ivy Biomedical Systems, Inc., Brandford, CT). Pulse pressure was calculated as previously described for supine and standing conditions [39]. Autonomic function testing [AFTs] included respiratory sinus arrhythmia [RSA] and Valsalva maneuver [VM]. Cardiac activity was recorded continuously using a single lead-II ECG and finger blood pressure was assessed non-invasively using a photoplethysmographic volume-clamp device (NOVA, Finapress Medical Systems, Netherlands). Adjustments for height differences between hand and heart levels were obtained using a hydrostatic height correction system. Finger blood pressure values were intermittently checked and cross-calibrated against brachial artery pressure using a clinical arm-cuff blood pressure device. AFTs recordings took place in a clinical tilt-test room, in a quiet environment with dim illumination. For RSA, subjects were recorded for 60 s baseline and were subsequently instructed to breathe at a fixed frequency (0.1 Hz) for 90 s. VM consisted in recording a baseline normal spontaneous breathing period of 60 s, followed by execution of a protracted (15 s) forceful expiratory effort against a resistance, producing an intrathoracic pressure increase between 30 to 40mmHg. After the effort, recording of additional 90 s recovery phase took place. Values that are considered physiological are RSA > 1.2 and Valsalva Ratio > 1.6 [40].

### Signals analysis

RSA and VM were analyzed offline using analysis software (Physiowave©, A. Diedrich, Vanderbilt University Medical Center, TN, USA). The RSA analysis took into account 90 s periods during a controlled 0.1 Hz breathing maneuver. The instantaneous maximal heart rate [HR] peaks and minimal HR valleys were automatically detected and the median HR_MAX_ and HR_MIN_ of the specific time period were calculated. RSA ratio was computed by dividing median HR_MAX_/ median HR_MIN_. Distinguished phases of VM (Baseline, VM1, VM2-early, VM2-late, VM3 and VM4) were identified as previously described [26] and the relative hemodynamic responses in terms of HR and systolic blood pressure [SBP] were automatically determined. In particular, the inter-phase ΔSBP indices that were computed were: (1) VM2e drop (lowest SBP in VM2-early – SBP Baseline) (2) VM2l rise (highest SBP VM2-late - lowest VM2e) and (3) VM4 overshoot (highest SBP VM4 – SBP Baseline).

Spectral analysis of continuous cardiovascular variables was performed offline using the BRS software (Physiowave© A. Diedrich, Vanderbilt University Medical Center, TN, USA) developed by one of the authors. R peaks and finger blood pressure waveform maxima were detected and displayed as R-R interval and SBP time series. The root mean square of standard deviations [RMSSD] and the standard deviations 1 and 2 [SD1 & SD2] were derived from Pointcare plots of consecutive R-R intervals [41]. The appropriate length (300 s) and stationarity of recorded segments for spectral analysis were defined following criteria previously described [42].

Periods of spontaneous breathing while supine were analyzed. Time series of R-R intervals [RRI] were interpolated in order to obtain a continuous signal as a function of time, low-pass filtered (cut-off frequency 0.5 Hz) and resampled at 4 Hz [42]. Linear trends were removed, and power spectral density was estimated with the FFT-based Welch algorithm. The power in the low (LF: 0.04 to < 0.15 Hz), and high (HF: 0.15 to < 0.40 Hz) frequency domains were calculated for R-R interval [LF_RRI_ and HF_RRI_] and SBP [LF_SBP_ and HF_SBP_].

### Statistical analysis

The primary aim was to assess for differences between LCP and healthy controls in HF_RRI_, which is considered a robust spectral marker of cardiac parasympathetic modulation [42–44].

Secondary endpoints were RSA, VM ratio, VM metrics, HRV indices obtained using linear and non-linear RR interval analysis and SBP variability indices.

A sample size calculation was performed taking into consideration expected modifications of the primary outcome using initial data of 7 LCP patients and 14 healthy controls (total N = 21 subjects), showing that it would be possible to detect a 200 ms^2^ difference in HF_RRI_ with type I error = 0.05 and 80% power. We analyzed 29 subjects in this study. Statistical analysis was performed using the commercially available PRISM software (GraphPad Software, LLC.). Shapiro-Wilk test was used to verify the normality of distribution for continuous variables. Also, a statistical assessment for potential outliers was performed using ROUT approach with a Q = 0.5%. Potential differences were assessed using Student’s t-tests or the Mann-Whitney test, depending on the individual dataset distribution. Statistical significance was reported using the coefficient α = 0.05. Values were reported as means ± standard error of the means [SEM]. One of the authors was granted full access to all the data reported in this manuscript and takes responsibility for its integrity and the data analysis.

## Results and discussion

### Subjects characteristics

15 females met diagnostic criteria for LCP and 16 healthy females without a history of COVID-19 infection were enrolled as controls. Past medical history and history of recent COVID-19-related illness are summarized in Table 1. The most common presenting symptoms included positional palpitations, lightheadedness, and dizziness. The mean ± SEM time from infection to development of symptoms was 44 ± 8 days. The mean ± SEM time from orthostatic symptoms onset to AFTs was 192 ± 25 days. Only one subject was hospitalized due to acute COVID-19 illness, for a total length of stay of 48 h. Two subjects (LC013 & LC014) were subsequently excluded from the initial analysis because of BMI > 35 Kg/m^2^.

**Table 1:** Long-COVID POTS past medical history and current clinical presentation

Patient	Covid illness	Infection-to-symptoms time			Symptoms at referral	Medications for Post-Covid at referral			Past medical history	Symptoms-to-AFTs time	
LC001	Fever, cough, dyspnea, nausea, abdominal pain		61 days	Palpitations, dizziness, dyspepsia			Verapamil	Reynaud			91 days
LC002	Dyspnea, chest tightness		31 days	Positional lightheadedness and dizziness			Metoprolol, Guanfacine	Asthma			153 days
LC003	Sore throat, rhinorrhea, dysgeusia,		31 days	Palpitations, exertional dyspnea			Metoprolol	GERD			93 days
LC004	Rhinorrhea, dyspnea, nausea		24 days	Palpitations			None	None			114 days
LC005	Cough		12 days	Palpitations, chest pain, positional dizziness, fatigue			None	Asthma			127 days
LC006	Fever, vomiting, diarrhea		35 days	Anorexia, mental clouding, fatigue, nausea			None	Asthma, MDD			217 days
LC007	Fever, dyspnea		92 days	Palpitations, dyspnea			Metoprolol	Hypothyroidism, PE, GERD			322 days
LC008	Fever		42 days	Orthostatic dizziness, presyncope			None	None			94 days
LC009	Fever, dyspnea		40 days	Palpitations, fever, diaphoresis			None	Pneumonia			349 days
LC010	Cough, palpitations		34 days	Palpitations			None	Glucose intolerance			96 days
LC011	Cough, fever, diarrhea		42 days	Palpitations			Propranolol	Cyclic mastodynia, DFS			256 days
LC012	Not specified		33 days	Palpitations, left-sided blurry vision			None	Anxiety, migraine			196 days
LC013	Dyspnea		57 days	Fatigue, chest pain, back pain			None	None			154 days
LC014	Not specified		28 days	Palpitations, presyncope			Metoprolol	Hypothyroidism, HTN, OSAS, kidney stones			240 days
LC015	Not specified		129 days	Fatigue, tinnitus, palpitations, extremities dysesthesias			None	None			374 days

Past medical history, onset and timeline of recent COVID19-related illness in the Long-COVID POTS group. *AFTs* indicates autonomic function tests, *GERD *Gastroesophageal reflux disease, *PE *Pulmonary embolism, *MDD *Major depressive disorder, *NSTEMI *Non-ST-elevation myocardial infarction, *DFS *Dermatofibrosarcoma, *HTN *Hypertension, *OSAS *Obstructive sleep apnea syndrome

Demographic and hemodynamic parameters are presented in Table 2. Both groups were similar in age and BMI. As expected, LCP had higher orthostatic deltaHR compared to controls (+ 40 ± 6 vs. + 21 ± 3, *p* = 0.004) and presented an orthostatic increase in SBP (+ 8 ± 4 vs. -1 ± 2, *p* = 0.04) that was not present in healthy controls. Also, pulse pressure was greater in the LCP population, both in supine (46 ± 2 vs. 39 ± 2, *p* = 0.02) and standing condition (44 ± 2 vs. 29 ± 2, p > 0.001).

**Table 2:** Demographic and hemodynamic characteristics

**Parameters**	**Long-COVID POTS**	**Healthy**	***P*** value	
	*N* = 13	*N* = 16		
Age (y)	35 ± 3	29 ± 2	0.69	
BMI (kg/m^2^)	26 ± 2	23 ± 1	0.26	
Baseline HR (bpm)	75 ± 2	68 ± 3	0.07	
Baseline SBP (mmHg)	115 ± 4	110 ± 3	0.39	
Baseline DBP (mmHg)	68 ± 3	71 ± 2	0.43	
Baseline PP (mmHg)	45 ± 2	39 ± 2	**0.02**	
Orthostatic ΔHR (bpm)	+ 40 ± 6	+ 21 ± 3	**0.004**	
Orthostatic ΔSBP (mmHg)	+ 8 ± 4	-1 ± 2	**0.04**	
Orthostatic ΔDBP (mmHg)	+ 10 ± 2	+ 9 ± 2	0.85	
Orthostatic PP (mmHg)	44 ± 2	29 ± 2	**> 0.001**	

Values are means ± SEM; *BMI* indicates body mass index, *HR *heart rate, *SBP S*ystolic blood pressure, *DBP *Diastolic blood pressure, *PP *Pulse pressure. *P* values were obtained using Student’s t-test or Mann-Whitney test.

### Autonomic function test

Cardiovagal assessment showed that LCP patients and healthy controls had similar VM ratio (1.67 ± 0.09 vs. 1.89 ± 0.07 *p* = 0.07) and RSA ratio (1.29 ± 0.03 vs. 1.27 ± 0.03 *p* = 0.42); however, 46.7% of LCP had VM ratio < 1.6 compared with 6.2% in controls (*p* = 0.01).

**Fig. 1:**
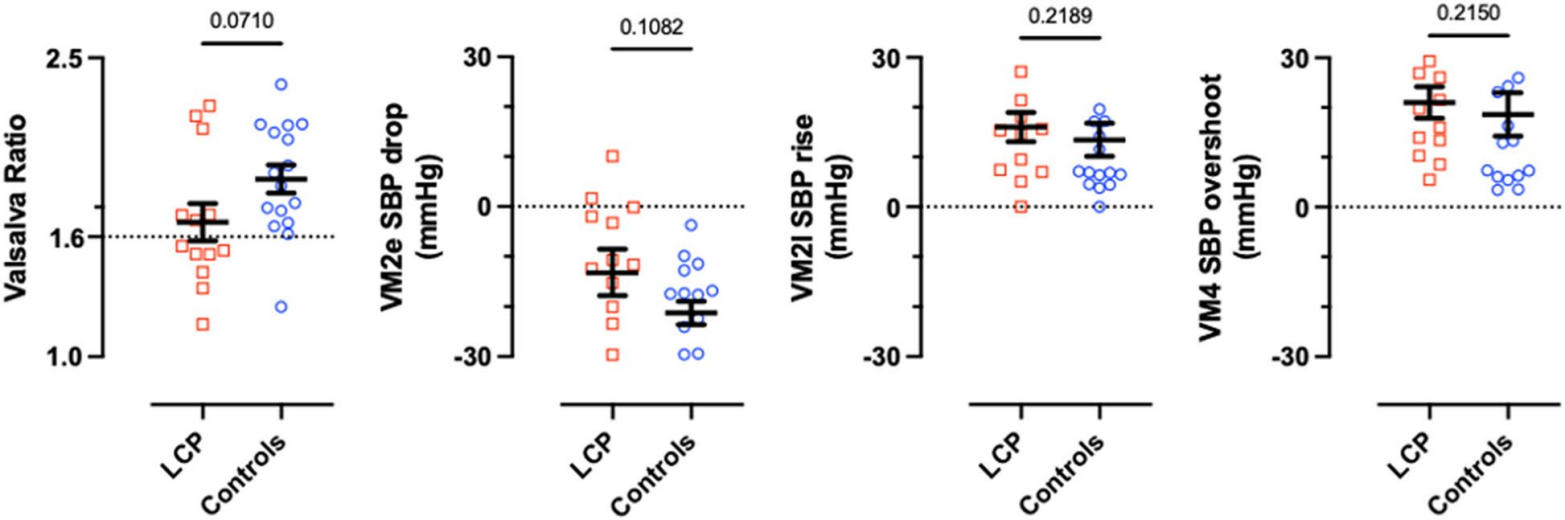
Valsalva ratio and Valsalva metrics analysis. The panel illustrates VM2e drop (lowest SBP in VM2e – SBP Baseline). VM2l rise (highest SBP VM2l - lowest SBP VM2e). VM4 overshoot (highest SBP VM4 – SBP Baseline). LCP indicates Long-COVID POTS. Values are means ± SEM. Significance values were obtained using Student’s t-test or Mann-Whitney test

VM hemodynamic changes during the four phases are presented in Fig. 1. LCP patients and controls had similar SBP during VM2e drop (-13.2 ± 4.6 vs. -21.3 ± 2.3 mmHg *p* = 0.11), VM2l rise (+ 16.0 ± 2.9 vs. + 13.5 ± 3.4 mmHg *p* = 0.22) and VM4 overshoot (+ 21.0 ± 3.2 vs. + 18.6 ± 4.4 mmHg *p* = 0.21). A tendency towards reduced mean VM SBP recovery times was found in the LCP group compared with controls (1.3 ± 0.3 vs. 5.0 ± 3.2 s, *p* = 0.07).

### Supine spectral analyses of BP and HR

Data from HRV analysis of LCP and healthy controls are reported in Table 3. The total power of spontaneous RRI variability was similar between the two groups (LCP 1671 ± 350 vs. healthy controls 2467 ± 544; *p* = 0.29). LCP patients, however, had reduced RMSSD (32.1 ± 4.6 vs. 48.9 ± 6.8 in healthy controls, *p* = 0.04) and HF_RRI_, both in absolute value (349 ± 105 vs. 906 ± 243 in healthy controls, *p* = 0.02) and in normalized units (32 ± 4 vs. 46 ± 4 n.u. in healthy controls, *p* = 0.02). Accordingly, the LF/HF ratio was increased in LCP (3.0 ± 0.7 vs. 1.3 ± 0.3 in healthy controls, *p* = 0.01). The SBP variability indices, on the other hand, were similar between LCP patients and healthy controls (LF_SBP_ 3.3 ± 1.0 vs. 4.6 ± 0.8 in healthy controls; *p* = 0.11 and HF_SBP_ 1.6 ± 0.4 vs. 1.8 ± 0.7 in healthy controls; *p* = 0.78).

**Table 3:** Supine spectral analysis of BP and HR variability in Long-COVID POTS and Healthy controls

Parameters	Long-COVID POTS	Healthy	*P* value
	*N *= 13	*N* = 16	
RMSSD	32.1 ± 4.6	48.9 ± 6.8	**0.04**
SD1	22.8 ± 3.3	34.2 ± 4.9	0.08
SD2	59.3 ± 5.4	67.7 ± 5.7	0.30
Total Power RRI	1671 ± 350	2467 ± 544	0.29
LF_RRI_ (ms^2^)	756 ± 174	982 ± 274	0.89
LF_RRI_ n.u.	66 ± 4	54 ± 4	0.06
HF_RRI_ (ms^2^)	349 ± 105	851 ± 253	**0.03**
HF_RRI_ n.u.	32 ± 4	46 ± 4	**0.02**
LF/HF	3.0 ± 0.7	1.2 ± 0.3	**0.01**
LF_SBP_ (mmHg^2^)	3.3 ± 1.0	4.6 ± 0.8	0.11
HF_SBP_ (mmHg^2^)	1.6 ± 0.4	1.8 ± 0.7	0.78

Values are means ± SEM. *RMSSD* indicates root mean square of standard deviations; *SD1* and *SD2*, Pointcare plots standard deviation 1 and 2; *RRI *R-R interval, *SBP *Systolic blood pressure, *LF *Low frequency bands, *HF *High frequency bands. *P* values obtained using Student’s t-test or Mann-Whitney test

## Discussion

Autonomic nervous system determinantion is traditionally complex and multimodal. A recent study from Novak et al. [45] used correlation-based network analysis including several autonomic and non-autonomic features (surveys, autonomic testing, sudoromotor testing, skin biopsy results, cerebral blood flow velocity, inflammatory markers) with the aim to investigate differences between POTS, PASC and controls. They found that PASC and POTS show positive correlations in nodes related to parasympathetic functions and inflammation, together with evidence of vascular dysfunction and altered cerebral blood flow. Although HRV and power spectral analysis do not account for global autonomic function determination, these techniques have been extensively used to characterize sympathetic and parasympathetic components of the autonomic nervous system. While evidence of long-term autonomic alterations in patients with PACS is accumulating [33–36] results are sometimes conflicting. The heterogeneity of PACS as currently classified may account for such differences. For this reason we aimed to study a specific subgroup of PACS patients, in particular those meeting criteria for LCP. Because of their characteristic manifestations they may especially be affected by altered cardiac autonomic modulation.

Initial clinical evaluation revealed that our LCP population was characterized by greater orthostatic HR increment and SBP increment. While these findings may be explained by cardiac and vascular sympathetic overdrive, further analysis of cardiovascular reflexes did not provide evidence of that. Instead, we found higher incidence of vagal impairment as assessed by Valsalva Ratio compared to healthy controls. Further, HRV analysis using different methodologies showed that supine RMSSD and HF_RRI_ were decreased in LCP. Altogether these results suggest that impaired cardiac parasympathetic activation may contribute to the pathophysiology of this novel syndrome.

The mechanisms by which SARS-CoV-2 infection may affect parasympathetic function is yet to be understood. Recent studies reported a neurotropic behavior in SARS-CoV-2, as the virus is known to replicate in neuronal cell cultures [46], and viral particles were found in human brain autopsies [7, 47]. Further, previous studies suggested that viral CNS invasion occurs through neurotropism, particularly in the cranial nerves innervating the rhino-oro-laryngopharyngeal mucosa (CNII, CNVII, CNIX, CNX). These gateways can grant access to neural structures located in the Frontal lobe and the Brainstem Pons and Medulla [47, 48]. In fact, human specimens of Pons and Medulla Oblongata have been found to express very high levels of ACE2 [49], the receptor for SARS-CoV-2 spike protein, and inhibition of ACE2 function has been shown to decrease vagal tone in animal models [50]. Therefore, viral infection and retrograde axonal transport to bulbar centers may theoretically affect important autonomic nuclei setting the conditions for impaired cardiovagal activity.

It is possible that impaired parasympathetic function in these patients could be deleterious not only by affecting cardiac modulation but also by altering systemic inflammatory responses through attenuation of the cholinergic anti-inflammatory reflex arc [51, 52]. Persistent inflammation has been described in SARS-CoV-2 survivors who have elevated IL-1β, IL-6 and TNFα levels [53], and our group previously reported increased inflammatory cytokine IL-6 plasma levels in POTS patients [54]. Of note, preliminary studies investigating the effects of transcutaneous auricular Vagus nerve stimulation in humans [44] reported an increase in HF_RRI_ and improvement in orthostatic and gastrointestinal symptoms in POTS.

This study has some limitations. It is a single center study with a relatively small number of patients enrolled. While elevated pulse pressure suggests that these patients are not hypovolemic, we did not measure circulating blood volume, which can be an important contributor to the clinical manifestations of LCP. However, hypovolemia would tend to increase sympathetic activity through physiological baroreflex compensation. We accounted for deconditioning by excluding prolonged hospital stay and severe disease in our study design, but the quantity and type of daily physical exercise was not systematically assessed and may be a confounder. We were not able to demonstrate significant alterations in sympathetic drive compared to healthy controls using blood pressure variability indexes and blood pressure response during VM, but we also did not include in this study robust measurements of sympathetic activity, such as muscle sympathetic nerve activity recordings. Importantly, we did not include in this study dynamic determination of autonomic functions by performing HRV and blood pressure variability analysis during head-up tilt testing. Future larger studies that include a more systematic characterization of central and peripheral sympathetic activity in LCP may clarify autonomic alterations that we were not able to detect. Our sample size was small, yet we had enough power to detect differences in the primary outcome.

## Conclusions

In summary, LCP is a new orthostatic intolerance syndrome characterized by exaggerated orthostatic tachycardia, chronic orthostatic intolerance, exercise intolerance, and fatigue. The prevalence of this condition is expected to increase in parallel with new SARS-CoV-2 infections, likely increasing its burden on US healthcare system. The pathophysiology and optimal clinical management for this condition are still unknown. However, our results suggest that patients with LCP may have abnormalities in cardiac parasympathetic regulation. These findings, if borne out by much larger studies, support the hypothesis that LCP is a different condition from POTS unrelated to SARS-CoV-2 infection, although the manifestations may be similar. Further, if future larger studies will confirm these results, patients affected by LCP may especially benefit from the use of treatments aimed at restoring parasympathetic modulation, such as electrical Vagus nerve stimulation techniques.

## Data Availability

The datasets used during the current study are available from the corresponding author on reasonable request.

## Abbreviations

AFTs: Autonomic function tests
HF: Power in high frequency bands
HR: Heart Rate
HRV: Heart rate variability
LF: Power in low frequency bands
PACS: Post-Acute COVID-19 Syndrome
POTS: Postural tachycardia syndrome
RMSSD: Root mean square of successive differences
RSA: Respiratory sinus arrhythmia
SBP: Systolic blood pressure
VM: Valsalva maneuver
RRI: R-R interval
LCP: Long-COVID POTS

## References

[1] Vosko I, Zirlik A, Bugger H (2023). Impact of COVID-19 on Cardiovascular Disease. Viruses 11 febbraio.

[2] Astin R, Banerjee A, Baker MR, Dani M, Ford E, Hull JH (2023). Long COVID: mechanisms, risk factors and recovery. Experimental Physiol gennaio.

[3] Siripanthong B, Nazarian S, Muser D, Deo R, Santangeli P, Khanji MY (2020). Recognizing COVID-19-related myocarditis: the possible pathophysiology and proposed guideline for diagnosis and management. Heart Rhythm settembre.

[4] Evans PC, Rainger GE, Mason JC, Guzik TJ, Osto E, Stamataki Z (2020). Endothelial dysfunction in COVID-19: a position paper of the ESC Working Group for Atherosclerosis and Vascular Biology, and the ESC Council of Basic Cardiovascular Science. Cardiovasc Res 1 dicembre.

[5] Jamal M, Bangash HI, Habiba M, Lei Y, Xie T, Sun J (2021). Immune dysregulation and system pathology in COVID-19. Virulence dicembre.

[6] Dani M, Dirksen A, Taraborrelli P, Torocastro M, Panagopoulos D, Sutton R (2021). Autonomic dysfunction in ‘long COVID’: rationale, physiology and management strategies. Clin Med gennaio.

[7] Aghagoli G, Gallo Marin B, Katchur NJ, Chaves-Sell F, Asaad WF, Murphy SA (2021). Neurological involvement in COVID-19 and potential mechanisms: a review. Neurocrit Care giugno.

[8] Jackson CB, Farzan M, Chen B, Choe H (2022). Mechanisms of SARS-CoV-2 entry into cells. Nat Rev Mol Cell Biol gennaio.

[9] Wang MY, Zhao R, Gao LJ, Gao XF, Wang DP, Cao JM (2020). SARS-CoV-2: structure, Biology, and structure-based therapeutics Development. Front Cell Infect Microbiol.

[10] Nalbandian A, Sehgal K, Gupta A, Madhavan MV, McGroder C, Stevens JS (2021). Post-acute COVID-19 syndrome. Nat Med aprile.

[11] van Kessel SAM, Olde Hartman TC, Lucassen PLBJ, van Jaarsveld CHM (2022). Post-acute and long-COVID-19 symptoms in patients with mild diseases: a systematic review. Family Pract 19 gennaio.

[12] Huang C, Huang L, Wang Y, Li X, Ren L, Gu X (2021). 6-month consequences of COVID-19 in patients discharged from hospital: a cohort study. The Lancet gennaio.

[13] Xiong Q, Xu M, Li J, Liu Y, Zhang J, Xu Y (2021). Clinical sequelae of COVID-19 survivors in Wuhan, China: a single-centre longitudinal study. Clin Microbiol Infect gennaio.

[14] Carfì A, Bernabei R, Landi F, for the Gemelli Against COVID-19 Post-Acute Care Study Group. Persistent Symptoms in Patients After Acute COVID-19. JAMA. 11 agosto 2020;324(6):603. 10.1001/jama.2020.12603 PMC7349096 32644129

[15] González-Hermosillo JA, Martínez-López JP, Carrillo-Lampón SA, Ruiz-Ojeda D, Herrera-Ramírez S, Amezcua-Guerra LM (2021). Post-Acute COVID-19 Symptoms, a Potential Link with Myalgic Encephalomyelitis/Chronic Fatigue Syndrome: A 6-Month Survey in a Mexican Cohort. Brain Sci..

[16] Shouman K, Vanichkachorn G, Cheshire WP, Suarez MD, Shelly S, Lamotte GJ (2021). Autonomic dysfunction following COVID-19 infection: an early experience. Clin Auton Res giugno.

[17] Parker AM, Brigham E, Connolly B, McPeake J, Agranovich AV, Kenes MT (2021). Addressing the post-acute sequelae of SARS-CoV-2 infection: a multidisciplinary model of care. Lancet Respir Med novembre.

[18] Novak P, Mukerji SS, Alabsi HS, Systrom D, Marciano SP, Felsenstein D (2022). Multisystem involvement in Post-Acute Sequelae of Coronavirus Disease 19. Annals of Neurology marzo.

[19] Liang L, Yang B, Jiang N, Fu W, He X, Zhou Y (2020). Three-month Follow-up Study of Survivors of Coronavirus Disease 2019 after Discharge. J Korean Med Sci..

[20] Cabrera Martimbianco AL, Pacheco RL, Bagattini ÂM, Riera R (2021). Frequency, signs and symptoms, and criteria adopted for long COVID-19: a systematic review. Int J Clin Pract ottobre.

[21] Johansson M, Ståhlberg M, Runold M, Nygren-Bonnier M, Nilsson J, Olshansky B (2021). Long-haul Post-COVID-19 symptoms presenting as a variant of Postural Orthostatic Tachycardia Syndrome: the swedish experience. JACC Case Rep aprile.

[22] Vernino S, Bourne KM, Stiles LE, Grubb BP, Fedorowski A, Stewart JM (2021). Postural orthostatic tachycardia syndrome (POTS): state of the science and clinical care from a 2019 National Institutes of Health Expert Consensus Meeting - Part 1. Auton Neurosci novembre.

[23] Shaw BH, Stiles LE, Bourne K, Green EA, Shibao CA, Okamoto LE (2019). The face of postural tachycardia syndrome - insights from a large cross-sectional online community-based survey. J Intern Med ottobre.

[24] Furlan R, Jacob G, Snell M, Robertson D, Porta A, Harris P (1998). Chronic orthostatic intolerance: a disorder with discordant cardiac and vascular sympathetic control. Circulation 17 novembre.

[25] Raj SR, Biaggioni I, Yamhure PC, Black BK, Paranjape SY, Byrne DW (2005). Renin-aldosterone paradox and perturbed blood volume regulation underlying postural tachycardia syndrome. Circulation..

[26] Jacob G, Diedrich L, Sato K, Brychta RJ, Raj SR, Robertson D (2019). Vagal and sympathetic function in neuropathic postural tachycardia syndrome. Hypertens maggio.

[27] Thieben MJ, Sandroni P, Sletten DM, Benrud-Larson LM, Fealey RD, Vernino S (2007). Postural orthostatic tachycardia syndrome: the Mayo clinic experience. Mayo Clin Proc..

[28] Miglis MG, Prieto T, Shaik R, Muppidi S, Sinn DI, Jaradeh S (2020). A case report of postural tachycardia syndrome after COVID-19. Clin Auton Res ottobre.

[29] Blitshteyn S, Whitelaw S (2021). Postural orthostatic tachycardia syndrome (POTS) and other autonomic disorders after COVID-19 infection: a case series of 20 patients. Immunol Res aprile.

[30] Jamal SM, Landers DB, Hollenberg SM, Turi ZG, Glotzer TV, Tancredi J (2022). Prospective evaluation of autonomic dysfunction in Post-Acute Sequela of COVID-19. J Am Coll Cardiol giugno.

[31] Ståhlberg M, Reistam U, Fedorowski A, Villacorta H, Horiuchi Y, Bax J (2021). Post-COVID-19 Tachycardia Syndrome: a distinct phenotype of Post-Acute COVID-19 syndrome. Am J Med dicembre.

[32] Raj SR, Arnold AC, Barboi A, Claydon VE, Limberg JK, Lucci VEM (2021). Long-COVID postural tachycardia syndrome: an american Autonomic Society statement. Clin Auton Res giugno.

[33] Marques KC, Silva CC, Trindade SDS, Santos MCS, Rocha RSB, Vasconcelos PFDC, Quaresma JAS, Falcão LFM. Reduction of Cardiac Autonomic Modulation and Increased Sympathetic Activity by Heart Rate Variability in Patients With Long COVID. Front Cardiovasc Med. 2022;9:862001. 10.3389/fcvm.2022.862001. 10.3389/fcvm.2022.862001 PMC9098798 35571200

[34] Stute NL, Stickford JL, Province VM, Augenreich MA, Ratchford SM, Stickford ASL (2021). COVID-19 is getting on our nerves: sympathetic neural activity and haemodynamics in young adults recovering from SARS‐CoV‐2. J Physiol 15 settembre.

[35] Shah B, Kunal S, Bansal A, Jain J, Poundrik S, Shetty MK (2022). Heart rate variability as a marker of cardiovascular dysautonomia in post-COVID-19 syndrome using artificial intelligence. Indian Pacing Electrophysiol J aprile.

[36] Asarcikli LD, Hayiroglu M, Osken A, Keskin K, Kolak Z, Aksu T (2022). Heart rate variability and cardiac autonomic functions in post-COVID period. J Interv Card Electrophysiol aprile.

[37] Sheldon RS, Grubb BP, Olshansky B, Shen WK, Calkins H, Brignole M (2015). 2015 heart rhythm society expert consensus statement on the diagnosis and treatment of postural tachycardia syndrome, inappropriate sinus tachycardia, and vasovagal syncope. Heart Rhythm giugno.

[38] Berlin DA, Gulick RM, Martinez FJ (2020). Severe Covid-19. Solomon CG, curatore. N Engl J Med 17 dicembre.

[39] Homan TD, Bordes SJ, Cichowski E, Physiology. Pulse Pressure. In: StatPearls [Internet]. Treasure Island (FL): StatPearls Publishing; 2022 [citato 19 marzo 2023]. Disponibile su: http://www.ncbi.nlm.nih.gov/books/NBK482408/. 29494015

[40] Robertson D. Primer on the Autonomic Nervous System [Internet]. Elsevier; 2012 [citato 17 febbraio 2022]. Disponibile su: https://linkinghub.elsevier.com/retrieve/pii/C20100651868.

[41] Shaffer F, Ginsberg JP (2017). An overview of Heart Rate Variability Metrics and norms. Front Public Health 28 settembre.

[42] Heart rate variability: standards of measurement, physiological interpretation and clinical use. Task Force of the European Society of Cardiology and the North American Society of Pacing and Electrophysiology. Circulation. 19961;93(5):1043–65. 8598068

[43] Malliani A, Pagani M, Lombardi F, Cerutti S (1991). Cardiovascular neural regulation explored in the frequency domain. Circulation agosto.

[44] Diedrich A, Urechie V, Shiffer D, Rigo S, Minonzio M, Cairo B (2021). Transdermal auricular vagus stimulation for the treatment of postural tachycardia syndrome. Auton Neurosci dicembre.

[45] Novak P, Giannetti MP, Weller E, Hamilton MJ, Mukerji SS, Alabsi HS (2022). Network autonomic analysis of post-acute sequelae of COVID-19 and postural tachycardia syndrome. Neurol Sci dicembre.

[46] Chu H, Chan JFW, Yuen TTT, Shuai H, Yuan S, Wang Y (2020). Comparative tropism, replication kinetics, and cell damage profiling of SARS-CoV-2 and SARS-CoV with implications for clinical manifestations, transmissibility, and laboratory studies of COVID-19: an observational study. Lancet Microbe maggio.

[47] Franca RA, Ugga L, Guadagno E, Russo D, Del Basso De Caro M. Neuroinvasive potential of SARS-CoV2 with neuroradiological and neuropathological findings: is the brain a target or a victim?. APMIS. 2021;129(2):37–54. 10.1111/apm.13092 33098147

[48] Baig AM, Khaleeq A, Ali U, Syeda H (2020). Evidence of the COVID-19 Virus Targeting the CNS: tissue distribution, Host–Virus Interaction, and proposed neurotropic mechanisms. ACS Chem Neurosci 1 aprile.

[49] Lukiw WJ, Pogue A, Hill JM (2022). SARS-CoV-2 infectivity and neurological targets in the brain. Cell Mol Neurobiol gennaio.

[50] Xu Z, Li W, Han J, Zou C, Huang W, Yu W (2017). Angiotensin II induces kidney inflammatory injury and fibrosis through binding to myeloid differentiation protein-2 (MD2). Sci Rep 21 marzo.

[51] Pavlov VA, Ochani M, Gallowitsch-Puerta M, Ochani K, Huston JM, Czura CJ (2006). Central muscarinic cholinergic regulation of the systemic inflammatory response during endotoxemia. Proceedings of the National Academy of Sciences..

[52] Martelli D, McKinley MJ, McAllen RM (2014). The cholinergic anti-inflammatory pathway: a critical review. Auton Neurosci maggio.

[53] Schultheiß C, Willscher E, Paschold L, Gottschick C, Klee B, Henkes SS (2022). The IL-1β, IL-6, and TNF cytokine triad is associated with post-acute sequelae of COVID-19. Cell Rep Med..

[54] Okamoto LE, Raj SR, Gamboa A, Shibao CA, Arnold AC, Garland EM (2015). Sympathetic activation is associated with increased IL-6, but not CRP in the absence of obesity: lessons from postural tachycardia syndrome and obesity. Am J Physiol Heart Circ Physiol..

